# Factors impacting the microbial production of eicosapentaenoic acid

**DOI:** 10.1007/s00253-024-13209-z

**Published:** 2024-06-11

**Authors:** Sérgio Sousa, Ana P. Carvalho, Ana M. Gomes

**Affiliations:** CBQF—Centro de Biotecnologia e Química Fina, Laboratório Associado, Escola Superior de Biotecnologia, Universidade Católica Portuguesa, Rua Diogo Botelho 1327, 4169-005 Porto, Portugal

**Keywords:** Fermentation engineering, Metabolic engineering, Downstream processing, Microalgae, Market products

## Abstract

**Abstract:**

The increasing applications for eicosapentaenoic acid (EPA) and the potential shortfall in supply due to sustainability and contamination issues related with its conventional sources (i.e., fish oils; seafood) led to an extensive search for alternative and sustainable sources, as well as production processes. The present mini-review covers all the steps involved in the production of EPA from microorganisms, with a deeper focus on microalgae. From production systems to downstream processing, the most important achievements within each area are briefly highlighted. Comparative tables of methodologies are also provided, as well as additional references of recent reviews, so that readers may deepen their knowledge in the different issues addressed.

**Key points:**

• *Microorganisms are more sustainable alternative sources of EPA than fish.*

• *Due to the costly separation from DHA, species that produce only EPA are preferable.*

• *EPA production can be optimised using non-genetic and genetic tailoring engineering.*

## Introduction

Human health status is strongly affected by diet. Beyond the lack of essential nutrients prevalent in many undeveloped areas, even in developed countries, where a balanced diet is available, other risk factors such as aging and pollution can lead to health problems. Therefore, the consumption of bioactive molecules that help the organism to maintain its health status is a key point to prevent the development of diseases. Indeed, recent advances have emphasized the importance of several bioactive molecules within human health. One of them is EPA.

Eicosapentaenoic acid (20:5; Fig. 1) is a long-chain polyunsaturated fatty acid (PUFA) from the *ω*3 family (i.e., those molecules in which the first double bond is in the position 3 when counting from its methyl end). Its configuration with 20-carbon unbranched carboxylic acid with five double bonds with a cis configuration provides the molecule with strong bioactive properties, such as those related with cardiovascular health benefits, including arrhythmia and stroke prevention, and blood pressure maintenance; additionally, EPA plays a role in the reduction of cholesterol and triglyceride levels in the body (Xia et al. 2020; Adarme-Vega et al. 2012). Furthermore, it is the precursor of several eicosanoids, a class of signalling molecules such as prostaglandins, leukotrienes, and resolvins, which play a role in inflammatory pathways, leading to a decrease in the production of pro-inflammatory cytokines (Jesionowska et al. 2023; Calder 2010). Finally, EPA is also a precursor of another *ω*-3 PUFA, docosahexaenoic acid (DHA 22:6) with important roles in neural and visual functions. Apart from the abovementioned roles in human metabolism, EPA is also very important in animal nutrition, namely aquaculture, among others.

**Fig. 1:**
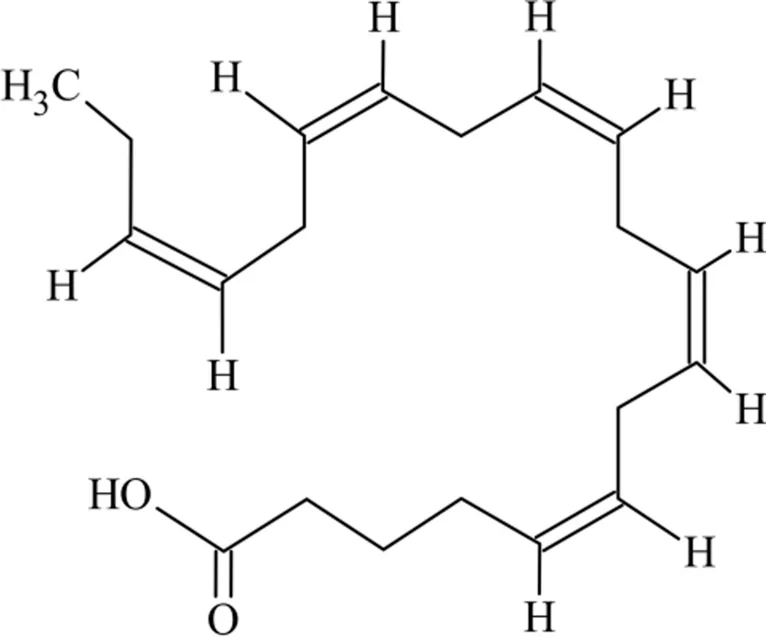
Eicosapentaenoic acid (EPA) structure

Despite EPA is not an essential fatty acid, as it is produced in human organism, the amounts produced are low (only 1–15% of its precursor, the alpha-linolenic acid is converted into EPA) and may become even lower due to conditions such as alcohol intake, smoking, poor exercise, and deficient diet. In fact, modern urban diets are low in *ω*-3 PUFA, and thus an extra intake through the diet is imperative. The recommended daily intake of EPA is usually provided together with DHA. Altogether, 250–500 mg/day is the suggested intake for the general health population, whereas the amounts rise to 700–1000 mg for pregnant and lactating women, and even higher than 1 g to treat medical conditions (Barta et al. 2021; GOED 2017; EFSA 2012).

These amounts are provided to human diet through the direct ingestion of fatty fish (e.g., sardine, mackerel, salmon, herring or tuna), foods enriched in EPA (functional foods), or supplements with rich-EPA oil (e.g., capsules). Current limitations on the use of fish as EPA source regard sustainability issues, seasonality, and potential contamination, especially when fish is caught in areas with environmental pollutants such as dioxins, methyl mercury, and polychlorinated biphenyls (Gerber et al. 2012). Additionally, the presence of a strong odor and off-flavors, combined to the high cost of obtaining an oil containing only EPA (since both EPA and DHA are present), adds disadvantages to this source.

To meet the recommended daily intakes of EPA, as abovementioned, ca. 0.7 million tonnes/year need to be produced only for human consumption, not to mention those for aquaculture market; this clearly points out the need of alternative sources which are simultaneously cost-effective and sustainable. An obvious choice is the use of microalgae, as they are the EPA primary producers in marine food chain. Indeed, the presence of EPA in fish is due to its consumption (directly or indirectly) of microalgae (phytoplankton) (Gu et al. 2021; Adarme-Vega et al. 2012). For example, sardine content in EPA and DHA may range from 1.2 to 22%, whereas the microalga *Nannochloropsis oceanica* produces EPA up to 42% of the total fatty acids (Xu 2022; Jovanovic et al. 2021).

Despite microalgal photoautotrophic production does not compete for potable water or nutrients, as opposite to the heterotrophic cultivation mode (Gu et al. 2021), biomass yields are lower. Nevertheless, although several microorganisms are able to heterotrophically produce DHA (e.g., *Crypthecodinium cohnii* or *Schizochytrium* sp.) (Barclay et al. 2010; Wynn et al. 2010), large-scale EPA production is usually dependent on phototrophic microalgae, which require light for growth (Sivakumar et al. 2022). Microalgal species which have been continuously reported as presenting high EPA contents (more than 25% of total fatty acids, corresponding to 3–5% EPA/dry cell weight) include *Monodus subterraneus*, *Odontella aurita*, *Chlorella minutissima*, *Phaeodactylum tricornutum*, and *Nannochloropsis oculata*, with the two latter as the most used in large-scale cultivations (Gu et al. 2021).

Current paths to enhance EPA production include genetic tailoring of microorganisms and photobioreactor engineering, the latter including the optimization of cultivation conditions (Sivakumar et al. 2022; Gu et al. 2021). Although both are important strategies to increase the intracellular EPA content and thus reduce production costs, the downstream processes are also an important step which must not be neglected.

**Fig. 2:**
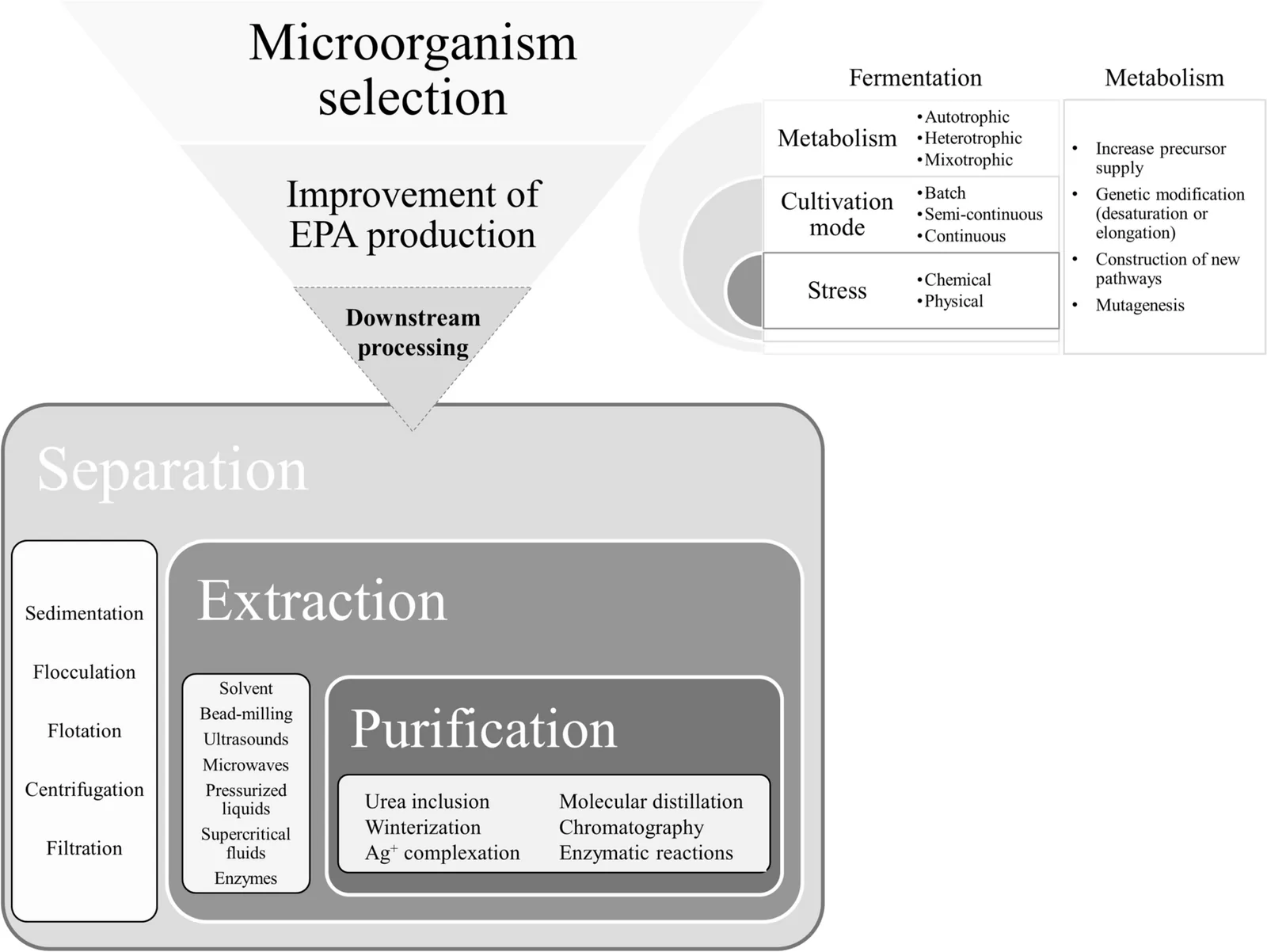
Overview of the steps involved in the production of EPA from a microorganism

Overall, the potential shortfall in EPA supply due to sustainability and contamination issues related with wild-caught fish promotes the research into alternative sources of production. The present overview intends to briefly highlight the most important achievements within this area, particularly regarding microalgae sources, from production systems to downstream processing, as depicted in Fig. 2. All the discussed areas will be enriched with recent references, to provide readers with sources where to deepen knowledge.

## Tools to improve EPA production in microalgal cultures

### Non-genetic tailoring/fermentation engineering

There are several ways by which the production of EPA by microorganisms (namely, microalgae) may be achieved. One of the first aspects that needs to be considered is how microalgae are going to be cultivated, namely in photoautotrophic, heterotrophic, or mixotrophic modes. In photoautotrophic growth, microalgae rely on light and carbon dioxide (CO_2_); this is the most utilized large-scale cultivation method, with open(raceway) ponds being the simplest and operationally cheapest systems (Abreu et al. 2022; Xia et al. 2020). However, biomass yield is low, the environmental conditions cannot (are hard to) be controlled, and microbial contamination may be an issue. Hence, the industry has looked at closed photobioreactors as alternatives, since growth conditions can easily be controlled, namely nutritional and environmental, as well as asepsis (Ren et al. 2022). Photobioreactors can have distinct configurations, being tubular, annular, or flat-plate the most commonly utilized (Sivakumar et al. 2022). Heterotrophic cultivation is another method that may be used, in which microalgae utilize organic compounds instead of light and CO_2_ (Ruiz et al. 2022). Although growth in these systems is faster and biomass reaches higher densities, costs are also higher, as is the potential for contamination by other detrimental microorganisms (Ren et al. 2022). A combination of both methods—mixotrophic cultivation, can also be utilized, wherein both metabolic traits are explored (Abreu et al. 2022). The selection of the best cultivation system toward EPA production must be assessed for the specific strain of microalga that is being utilized.

The cultivation process of microalgae can also be classified in terms of how the nutrients are “delivered.” The process can be divided into batch, semi-continuous (repeated-batch or fed-batch) and continuous. The first is the most widely utilized and consists in a system in which throughout growth no alterations to the medium (additions or removals of nutrients or products) are performed, which can eventually lead to growth inhibition by depletion of nutrients and/or formation of detrimental metabolites. Regarding semi-continuous systems, in repeated batch, at determined time intervals, a part of the culture is completely substituted by fresh medium, while in fed-batch substrates are added to the growth medium in the necessary amounts to avoid their depletion (growth will cease when high biomass amount impedes light availability). In continuous systems, in addition to substrate supplementation, removal of metabolic detrimental compounds is also performed (Sivakumar et al. 2022).

### Stress factors (chemical or physical)

As Borowitzka (2018) clearly explained, the word “stress” is used by different authors to describe distinct (chemical or biological/metabolic) processes. Hence, herein, the biotic and abiotic conditions that impact growth will be designated “stress factors.” It has long been known that, when a nutritional, or environmental, condition is below or above the optimal for microbial growth, the cells react by changing their metabolism, which in some cases can lead to alterations in lipid profile and consequently, in EPA amount. These metabolic traits are often utilized as tools to increase the EPA contents in microalgae cultures. The stress factors which are usually applied can be of chemical or physical nature.

#### Chemical

The most utilized chemical stress factor for enhancing lipid and EPA contents in microalgae is nitrogen starvation/limitation/depletion. In response to low amounts of nitrogen in the growth medium, microalgae redirect their metabolism from growth to the production and accumulation of storage compounds such as triacylglycerols (TAG) (Sousa et al. 2023a). Phosphorous starvation has also been applied to increase microalgae lipids, similarly, increasing TAG amounts (Paliwal et al. 2017). However, PUFA (EPA included) are not present in most microalgae TAG, which production can be achieved at the expense of PUFA and, therefore, limitation of those nutrients may not be a suitable strategy to increase EPA content (Sousa et al. 2023a). Nonetheless, nitrogen starvation has been reported to increase EPA content in some microalgae (Paliwal et al. 2017). Salt-induced stress can also be utilized, since osmotic stress can originate metabolic changes, namely regarding membrane fluidity, which is correlated with unsaturation degree (amount of PUFA) and, therefore, can influence EPA contents (Sajjadi et al. 2018).

#### Physical

Temperature and light are the physical stress factors most commonly associated with lipid alterations in microalgae. Low temperature is known to be an effective EPA-enhancing strategy in microalgae cultures since, as a response mechanism, they increase the unsaturation degree of the membrane (through the increase of PUFA) to increase its fluidity (Chen et al. 2017; Minhas et al. 2016). Concerning light as stress factor, two strategies are possible, namely light intensity or light quality/type (Sousa et al. 2022). Low-light intensity has been associated with higher EPA contents, possibly resulting from the need to increase photosynthetic capacity, which may be achieved through an increase in thylakoid membranes (Paliwal et al. 2017; Minhas et al. 2016). Light quality/type also impacts lipid content of microalgae, and it was reported that specific wavelengths can be used to increase EPA amounts (Sousa et al. 2022). Other physical stress factors include pH, which may result in an increase of membrane saturated fatty acids with the goal of decreasing membrane fluidity, but is also reported to increase TAG accumulation and, thus, can increase EPA in microalgae in which it is present in those molecules (Sajjadi et al. 2018; Chen et al. 2017; Paliwal et al. 2017). Radiation also has the potential to impact lipids and, in fact, increased PUFA and EPA contents were achieved by using UV-A radiation (Sousa et al. 2023a; Paliwal et al. 2017).

### Genetic tailoring/metabolic engineering

Metabolic engineering has been widely explored to increase EPA contents in microalgae and other microorganisms. The concept is to, by understanding the cellular pathways, be able to genetically modulate the production of a specific compound, namely EPA.

One strategy employed regards the improvement of EPA precursor supply, which can be achieved by increasing the expression of the acetyl-CoA synthase, that is responsible for the synthesis of acetyl-CoA from acetate. Since the de novo biosynthesis of EPA main precursor is acetyl-CoA, EPA will ultimately be increased by the acetyl-CoA synthase higher expression (Behera et al. 2021; Xia et al. 2020). Genetic modification of elongation and desaturation pathways can also result in EPA production enhancement, as in the case of the overexpression of Δ17 desaturase, which is an enzyme in the pathway by which EPA is synthesized (Xia et al. 2020). Alternatively, as performed by DuPont, pathways can be constructed in model microorganisms (in that specific case, *Yarrowia lipolytica* yeast), by insertion of a number of different genes, which enabled the production of EPA through the overexpression of specific enzymes of an EPA producing pathway (namely, Δ6 pathway) (Xie et al. 2015). Mutagenesis is also a mechanism/phenomenon by which microorganisms with increased EPA production may be achieved. Mutagenesis can be physical (e.g., radiation) or chemical (e.g., ethyl methane sulphonate), mutating the genome of the microorganism, or it can be insertional, in which external DNA is inserted into the microorganism and subsequently integrated in its genome (Jakhwal et al. 2022).

As described, there is a multitude of tools that may be utilized to improve EPA production by microorganisms. For a more extensive description of the methodologies and their underlying mechanisms, Abreu et al. (2022), Jakhwal et al. (2022), Paliwal et al. (2017), and Minhas et al. (2016) can be consulted.

## Downstream processing

Downstream processing is the step of the production process that represents the largest economic burden. It presents several challenges and includes procedures that are (can be) major bottlenecks in the overall production of EPA. As a result, and also due to the growing interest in the sustainability of the process, review articles in recent years have increasingly focused on downstream processing in its entirety. This step can be divided into three distinct stages, namely separation (harvest biomass), extraction (collect the lipid fraction), and purification (isolate EPA).

### Separation (harvest)

**Fig. 3:**
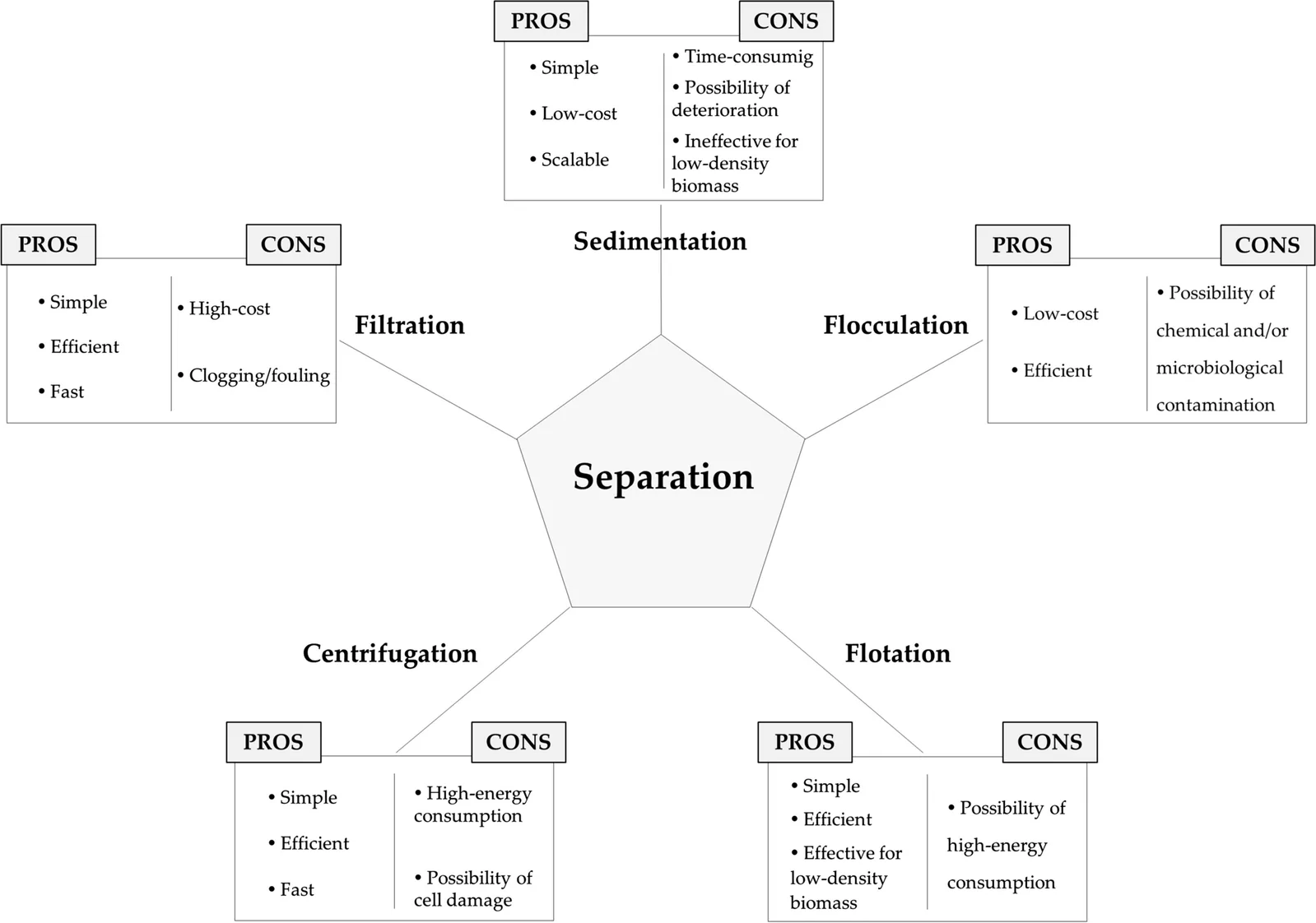
Separation methods positive and negative traits

Following the production of EPA by microorganisms, a succession of steps/procedures must be undertaken toward its final obtainment in a purified form. The first step consists in separating and collect the EPA-containing biomass from the medium in which the microorganism was grown. To achieve such goal, a multitude of techniques/technologies can be employed, which have been reported to account for as much as 40% (Jakhwal et al. 2022 Jia et al. 2022). As in every process, the different methodologies that can be employed have their strengths and weaknesses (Fig. 3).

Among the distinct harvesting techniques, sedimentation and flocculation are the ones that entail the lowest costs. Sedimentation relies on the deposition of the biomass through the force of gravity (Ren et al. 2022; Barta et al. 2021; Yin et al. 2020). Consequently, harnessing biomass through this methodology is slow, and it may even be ineffective if the microorganism has a low mass (low density) (Ren et al. 2022; Yin et al. 2020). Moreover, due to such timespan, there is the possibility of deterioration and/or contamination of the biomass (Barta et al. 2021; Yin et al. 2020). However, the method is simple and requires minimal equipment and operational costs, thereby making it economically appealing (Barta et al. 2021; Japar et al. 2017).

Flocculation is a method in which there is an aggregation of the cells, leading to the formation of larger complexes that enables their collection easier. The method is based on electrostatic interactions that, in normal conditions, repel the negatively-charged cellular surfaces from their counterparts (thereby preventing aggregation), but in the presence of flocculants are reduced or abolished (Barta et al. 2021; Patel et al. 2020; Shen et al. 2013). There are several types of flocculation, namely auto-, bio-, chemical, and physical flocculation (Laamanen et al. 2021; Enamala et al. 2018; Japar et al. 2017). Aluminium, ferric, and zinc salts are commonly used for flocculation (Patel et al. 2020), as well as natural polymers, such as chitosan and plant-derived compounds (Laamanen et al. 2021; Liber et al. 2020). One environmental condition that can induce flocculation is the increase in pH of the growth medium (Enamala et al. 2018; Japar et al. 2017). The aggregation of cells may also be achieved biologically, via contact with other microorganisms, and by employing physical principles such as hydrodynamics or electric charges (Ren et al. 2022; Barta et al. 2021; Liber et al. 2020; Enamala et al. 2018). Flocculation can be utilized in combination with other methods, such as sedimentation or flotation, to increase the biomass yield (Laamanen et al. 2021; Japar et al. 2017). The main advantages of the method are the efficiency and low cost, while a major (and worrisome) drawback is the possibility of chemical or microbiological contamination (Yin et al. 2020; Japar et al. 2017), as well as high-energy requirements when electrochemical flocculation is concerned (Laamanen et al. 2021).

Another method utilized to harvest biomass is flotation, which consists in injecting a gas (typically air or ozone) into the growth medium, which generates bubbles that, when rising to the surface, transport/carry the microorganism. Once on the surface, biomass can easily be collected through skimming (Ren et al. 2022; Laamanen et al. 2021; Suparmaniam et al. 2019). This method may be combined with flocculation, which will aggregate the cells prior to the elevation via the gas bubbles (Laamanen et al. 2021). Flotation can be quite effective for collecting low-density microorganisms such as microalgae (Laamanen et al. 2021; Patel et al. 2020; Yin et al. 2020). Concerning negative aspects, not being cost-efficient is the main disadvantage mentioned (Ren et al. 2022; Patel et al. 2020); however, since there are distinct flotation methodologies, contradictory reports can be found according to the strategy employed (Laamanen et al. 2021; Yin et al. 2020; Suparmaniam et al. 2019).

Centrifugation relies on the centripetal force exerted by high-speed rotations, which leads to the separation of the two phases (liquid–medium; solid–biomass) and can also be applied (Yin et al. 2020; Suparmaniam et al. 2019; Enamala et al. 2018). There are several types of centrifuges available for industrial application, such as hydrocyclones, decanters, disk stack, and spiral (Barta et al. 2021; Laamanen et al. 2021; Japar et al. 2017). Separation through centrifugation is highly effective and fast, although the process requires high-energy consumption, and the high shear forces applied to the cells can result in damage of their structural integrity and rupture, consequently leading to leakage of compounds (Laamanen et al. 2021; Yin et al. 2020; Suparmaniam et al. 2019).

Lastly, separation can also be achieved by filtration, in which membranes are utilized to retain the biomass, while the growth medium flows through (Barta et al. 2021; Yin et al. 2020). There are several types of membranes, as well as distinct processes (Barta et al. 2021; Liber et al. 2020; Yin et al. 2020). The method has high recovery efficiency, but high costs due to the price of the filters and the need to substitute them as a result of clogging/fouling, which also limits the flow and, consequently, the volumes that can be treated/filtered (Laamanen et al. 2021; Liber et al. 2020; Yin et al. 2020).

A careful comprehensive analysis, on a case-by-case basis, must be undertaken when aiming to harvest a specific microorganism for EPA production. It must be pointed out that the recovery of water from culture media is an important parameter under study in the last years, not only because of the economic benefits resulting from reducing the total amount of water necessary, but also due to sustainability considerations. Rather complete comparisons between the distinct harvesting methods previously described, including extensive analysis of flocculants and flocculation mechanisms, can be found in Laamanen et al. (2021), Yin et al. (2020), and Suparmaniam et al. (2019).

### Extraction

Once biomass has been collected, the next step is to extract the lipophilic fraction/compounds, in which lipids (including EPA) are comprised. Throughout the years many organic solvents (such as chloroform, hexane, and dichloromethane), and combinations thereof, have been explored with the aim of obtaining the highest possible yields. Nowadays, there is a growing concern in finding alternative, less health and environmental hazardous, solvents (sometimes designated “green” solvents) to the traditionally utilized organic ones. Since, usually, such solvents present lower yields, distinct technologies are also being applied, in order to increase their extraction efficiencies (Sousa et al. 2023b). As for separation, there is also a panoply of technologies that can be utilized for the extraction lipids from cells, which also have advantages and disadvantages (Table 1), according to the technology and the mechanisms by which extraction is performed.

**Table 1:** Advantages and disadvantages of the most cited cell disruption and lipid extraction methodologies

Cell disruption and lipid extraction method	Advantages	Disadvantages
Soxhlet	Simple	Slow
		Toxic/hazardous organic solvents
Folch/	Simple	Toxic/hazardous organic solvents
Bligh & Dyer	Fast	
Bead-milling	Efficient	Difficult scale-up
	Simple	Heat generation
	Fast	High-energy consumption
High-pressure homogenization	Efficient	High-energy consumption
	Low temperature	Possibility of emulsification
Ultrasounds	Low temperature	Possible degradation of compounds
	Selective	High-energy consumption
	Cost-efficient	Difficult scale-up
Microwaves	Fast	High-energy consumption
	High yields	Specific solvents required
	Low solvent volume	Heat-generation
Electric fields	Mild conditions	Aquisition and maintenance costs
	Low energy	Low efficiency
Pressurized liquids	Fast	High-cost
	Low solvent volume	Detrimental processing conditions
Supercritical fluids	Efficient	High-cost
	Fractioning possible	Low polarity
Acid/alkaline lysis	Efficient	Degradation of sensitive compounds
	Simple	
Enzyme-assisted	Specific	High-cost Processing time
	Mild conditions	
	Easely-scalable	
Ionic liquids/deep eutetic solvents	Low temperature	Potential chemical contamination (sample and environment)
	Physical and chemical properties easely adjusted	

The aim of these technologies is to facilitate the access of the extraction solvent to the compound intended to extract. Generally, extraction technologies can be divided by the principle/mechanism by which they exert their action, and in this sense, technologies can be classified as being of physical (including mechanical), chemical, or enzymatic nature (Chen et al. 2023; Sousa et al. 2023b; Ren et al. 2022). Additionally, there are also some more “unconventional” methods. Many of those methods aim to compromise or rupture the cell wall of the EPA-containing microorganism, thus allowing/facilitating the extraction.

The older, more basic, methods for lipid extraction are the solvent-based, including the traditional Soxhlet, the Folch et al. (1957), and the Bligh and Dyer (1959) methods, which utilize organic solvents (Zhou et al. 2022; Imbimbo et al. 2020; Chua and Schenk 2017).

Concerning the physical-based methods, these include (as aforementioned) mechanical methods, such as pressing (Zhou et al. 2022; Menegazzo and Fonseca 2019), bead-milling (Mehariya et al. 2021; Li et al. 2019), and high-pressure homogenization (Chen et al. 2023; Ren et al. 2022), which rely on mechanical forces to rupture the microbial cells. Ultrasounds (sonication) and microwaves are two physical-based methods that are sometimes also classified as mechanical, since the phenomenon generated by these methods results in a “mechanical” force being exerted. In ultrasounds-assisted extraction, the propagation of the ultrasonic waves through the liquid creates bubbles which eventually collapse due to pressure differences (this phenomenon is designated cavitation), and the shear forces resulting from the bursting of the bubbles lead to the cellular rupture (Sousa et al. 2023b; Zhou et al. 2022; Enamala et al. 2018). Microwave-assisted extraction is based on the evaporation of water from the cells, due to heat generated by the application of the microwaves, which generates forces from within the cell and result in their rupture (Chen et al. 2023; Mehariya et al. 2021). Other physical-based extraction methods are based on electric fields, such as pulsed electric fields, in which the application of electric fields leads to what is called electroporation (creation of pores or enlargement of existing ones in the cell wall by applying a strong enough electric field, thus increasing solvent penetration and consequent extraction). Electroporation can be reversible or irreversible, depending on the intensity of the electric field (Mehariya et al. 2021; Geada et al. 2018). Physical properties of solvents can also be explored, as in the cases of supercritical fluid extraction, in which solvents are utilized above their critical pressures and temperatures, and pressurized liquid extraction, in which the pressure and temperature are high but do not surpass their critical points, and thus the solvents are maintained liquid throughout the extraction (Sousa et al.2023b; Imbimbo et al. 2020).

Alkaline or acid lysis are chemical-based extraction methods in which such substances are utilized to promote cell rupture (Raj et al. 2023; Ren et al. 2022). Osmotic shock is based on the osmotic pressure from differences in salt concentration between the inside and outside of the cells, which result in turgidity or plasmolysis (according to the surrounding medium being hyper- or hypotonic, respectively), and both phenomena ultimately result in leakage (Chen et al. 2023; Menegazzo and Fonseca 2019). Ionic liquids and deep eutetic solvents are types of solvents that have been studied as green alternatives. Ionic liquids are composed of anions and cations and consist of salts that are liquid (molten) at temperatures below 100° (some even at room temperature), and which characteristics, such as polarity and viscosity, can be manipulated. Deep eutetic solvents are mixtures of hydrogen-bond acceptors and donors, with low-melting temperature, being biodegradable, presenting low toxicity and a simple and cost-efficient synthesis (Raj et al. 2023; Sousa et al. 2023b; Imbimbo et al. 2020).

Another type of methodology is enzyme-assisted extraction, in which enzymes are utilized to breakdown/disintegrate the cell wall components, thereby providing access of the solvent to the compound to be extracted. This method is performed at mild temperature conditions, which is a positive trait when heat-sensitive compounds such as EPA are to be extracted. However, the cost of the enzymes is a negative aspect when looking to apply the method at an industrial scale (Zhou et al. 2022; Menegazzo and Fonseca 2019; Zhang et al. 2019; Phong et al. 2018).

Besides the extraction methods previously described, there is a panoply of others, some more unconventional than others. Some examples of such extraction methods include supramolecular solvents (Salatti-Dorado et al. 2019), CO_2_ explosion (Günerken et al. 2019), antibiotics to lyse Gram-negative bacteria (Patel et al. 2020), viral cell lysis (Cheng et al. 2013), and autolysis (Halim et al. 2019). When extractions methods are applied at an initial stage, after which the use of extraction solvents is still required, they can be (and are often) designated as pretreatments.

Analyzing all the extraction methods described above, one can conclude that, similarly to the stated regarding harvesting methodologies, many have the potential to be used to obtain EPA from microorganisms. Nonetheless, to be able to industrially implement one, several factors must be considered so that economic viability can be achieved. A more in-depth analysis of the extraction mechanisms described, as well as examples of their utilization can be found in Sousa et al. (2023b), Zhou et al. (2022), and Zhang et al. (2019).

### Purification

Most of the EPA-purification methods reported and utilized nowadays aim to enrich its content within the lipid fraction, i.e., to concentrate EPA by removal/separation of other compounds from the extract. Hence, hereon methods used to concentration/purification of *ω*3 PUFA (EPA and DHA) will be discussed; the non-specificity among these *ω*3 PUFA relies in the fact that their similar chemical structure restrains an effective separation with most of the methodologies commonly employed.

The most used method is urea inclusion/complexation/adduction, a simple method which is able to separate PUFA from mono- and unsaturated fatty acids (Chen et al. 2023; Sivakumar et al. 2022; Li et al. 2019). The method is based on the formation of crystal inclusion complexes between urea and mono- and unsaturated fatty acids during crystallization of urea, a phenomenon which does not occur with PUFA due to its spatial configuration, more specifically, the lack of linearity due to the number of double bonds (Chen et al. 2023). Hence, following cooling leading to crystallization, a simple filtration can be performed, and the remaining liquid will be PUFA-enriched (Sivakumar et al. 2022; da Silva et al. 2019).

Winterization, or low-temperature crystallization, relies on differences in melting points of distinct degrees of saturation of fatty acids. Saturated fatty acids melting temperatures are higher than those of unsaturated ones, which results in unsaturated fatty acids being liquid at temperatures at which saturated fatty acids are still solid (Chen et al. 2023; da Silva et al. 2019). Moreover, solubility in organic solvents is also affected by temperature, and unsaturation favors solubility. Consequently, when decreasing the temperature, due to those two phenomena, saturated fatty acids solidify before the unsaturated, which allow for their separation through a solid–liquid separation (Chen et al. 2023).

Other methods include silver complexation, in which the doble bonds of unsaturated fatty acid form complexes with Ag^+^, which are then separated through other methods (such as chromatography or membranes) (Chen et al. 2023). Fractional (molecular) distillation utilizes temperature and pressure to manipulate the molecular mean free path and consequently, the evaporation rate of fatty acids with distinct degrees of unsaturation, specifically, to evaporate faster the ones with lower unsaturation (higher molecular mean free path) (Chen et al. 2023). Chromatographic methods, such as high-performance countercurrent chromatography (Bárcenas-Pérez et al. 2022), silica-gel chromatography (Sánchez et al. 2022), or supercritical fluid chromatography (Li et al. 2019), as well as lipase enzymatic reactions, in which lipase selective hydrolysis can promote the enrichment in EPA (Wang et al. 2023; da Silva et al. 2019), have also been applied to concentrate/purify EPA.

Table 2 presents advantages and disadvantages of some of the methods described as suitable for EPA concentration/purification.

**Table 2:** Advantages and disadvantages of EPA purification methods

Method	Advantage	Disadvantage
Urea complexation	Simplicity	Difficulty in separating similar unsaturations
	Low-cost	
Winterization	Simplicity	Low-efficiency
	Low-cost	Large amount of solvents
Silver complexation	Separation efficiency	High-cost
	Product purity	Heavy metal contamination
Molecular distillation	Mild conditions	High-energy consumption
	Possibility of continuous production	Low-purity

A common characteristic of most of the methods described is that separation is achieved based on unsaturation degree. Consequently, due to the similarity between EPA and DHA, most are unable to separate these two fatty acids.

A more detailed explanation of the mechanisms of some of the methods previously described can be found in a recent review by Chen et al. (2023), concerning hotspots and production techniques of PUFA from microalgae.

## Industrial production and market value

Scientifically established evidence of the wide range of positive clinical outcomes of EPA and its resulting bioactive metabolites on human health promotion and lifestyle diseases prevention, coupled to regulatory flexibility, and authorization has brought this ω3 fatty acid to the attention of food (including pet foods), nutraceutical and pharmaceutical markets, worldwide, becoming a part of consumer’s daily diets as a preventive healthcare measure.

The increasing applications for EPA and its finite conventional sources have led to an extensive search for alternative sources, including microalgae, lower fungi, and marine bacteria; the latter few, in particular the highly biodiverse microalgae, have been gaining prominence over the past years as a sustainable EPA source with less organoleptic (taste/aroma) barriers than fish oils. EPA from cultured microalgae is cholesterol-free and contaminant-free, and offers an acceptable taste and enables attractive labels such as “vegetarian/vegan,” “kosher,” and “organic,” important differences when compared to fish-derived EPA. From a commercial standpoint, production of oils rich in ω3 (EPA/DHA) from microalgae have been a major development focus of this century (Winwood 2013), and those that have reached commercialization stage, even though at a smaller scale than those derived from fish, have a higher market value. Their use in infant formulae, functional fortified foods and beverages, dietary supplements, and pharmaceuticals formulation is already well established worldwide from Asia–Pacific (which holds the major share in ω3 fatty acids products), to North and South America (growing market driven by investments in innovative technologies and high consumption of fortified products), to Europe, Middle East, and Africa with less market share. The higher prices associated with concentrates with EPA and DHA concentrations of up to 90% are used particularly within the nutraceutical and pharmaceutical markets corresponding to the largest market segment in terms of market value (van der Voort et al. 2017).

Indeed, several market reports reveal a shift in consumer demand for dietary supplement sources instead of functional foods in countries and regions like the United States, China, and Europe. In Europe, the rise in the geriatric population may account for the significant CAGR of 11.50% to be witnessed in the EPA market during the forecast period of 2022–2029 (DataBridge Market Research 2022). Another recently published market report estimated the EPA (and DHA) market at USD 1.74 billion in 2023 and forecasted a continuous growth to values of USD 2.41 billion market to be reached by 2028, growing at a CAGR of 6.76% during the forecast period (2023–2028) (Mordor Intelligence 2023). Currently, there are more than a dozen main producing companies worldwide involved in the research, development, manufacturing or marketing of EPA alone, or in co-production with DHA, as listed in Table 3.

**Table 3:** Available commercial producers and corresponding products, source, and EPA and DHA concentrations

Producer (country)	Products	Source	Concentration	Online source
DSM (Netherlands)	Life’s Omega	Algal oil (*Schizochytrium* strain)	150 mg EPA + 300 mg DHA	https://www.dsm.com/content/dam/dsm/human-nutrition/pdfs/dsm-lifesomega-brochure.pdf https://www.dsm.com/human-nutrition/en/products/nutritional-lipids/lifes-limitless-algal-sourced-omega-3.html
Lipa Pharmaceuticals	Almega PL	Algal oil (*Nannochloropsis oculata*)	EPA (25%)	https://www.lipa.com.au/images/Exclusive_Materials/Compressed_PDFs/Key_Material_Booklets_New_Covers/Almega_PL_Alternate_Sell_Sheet_compressed.pdf
BASF SE (Germany)	Maxomega EPA 96/97 EE	Fish oil	Min. 96% (w/w) EPA EE	https://virtualpharmaassistants.basf.com/s/product?recordId=01t2p00000AiBHSAA3
	PronovaPure® 500:200 EE	Fish oil	Min. 500 mg EPA/g; 200 mg DHA	https://pharma.basf.com/products/pronovapure-500200-ee
BTSA Biotecnologias Aplicadas S.L. (Spain)	Biomega-Tech® Fish	Fish oil	Up to 70% EPA	https://www.btsa.com/en/marcas/biomega-tech-fish/
Epax (Norway)	Epax Ultra Concentrate EPAX 5520 EE	Marine origin	550 mg EPA 200 mg DHA	https://www.epax.com/products/epax-omega-3/
Croda International plc (UK)	Incromega™ EPA 700E-LQ-(LK)	Marine origin	700 mg EPA	https://www.crodapharma.com/en-gb/product-finder?marketapplication=6&currentPage=1&pageSize=20&sortBy=recommended&lang=en-gbadm-omega-3-product-sheet-eng-na-20-final.pdf
Archer Midlands Company (USA)	Onavita Omega-3 EPA/DHA blends	Algal origin	NA	
Farbest Brands (USA)	ZMC Omega-3 4030EE	Fish oil	40% EPA 30% DHA	https://farbest.com/products/zmc-omega-3-fish-oil-4030ee-40-epa-30-dha/

*EE* ethyl ester

The EPA production potential of microalgae depends on the characteristics of the specific algal species and the cultivation strategies developed, as explored in the previous section of this review. The production potential of microalgae has been optimised in terms of oil yield maximisation using different sustainable production strategies, from fermentation to metabolic engineering as recently reviewed by Xia et al. (2020), Jakhwal et al. (2022), and Kumari et al. (2023)—who focused specifically on non-genetic and genetic tailoring methods to enhance sustainable, renewable, EPA production.

Although algal oil production is expensive, economies of scale have attenuated the final cost, and the gap between fish oil and algal oil costs has been continuously narrowing over the more recent years.

The major advances in upstream and downstream bioprocessing of algal oils were covered in the previous section and further comprehensive reviews tackling the different strategies were also provided. Algal sources of ω3 rich oils are increasingly meeting manufacturer’s needs both in terms of concentration and sustainable extraction processes, especially in what concerns DHA. In the case of EPA synthesis, the complex and less energetically efficient production systems for the different microalgae selected as cell factories still hinders market growth. Nonetheless, emerging biotechnological strategies to attain improved production of EPA are being exploited to provide promising solutions for industrial applications. For example, by multiplying the gene encodings responsible for PUFA synthase (PFA1, PFA2, PFA3), DSM has developed recombinant microalgae, from the *Schizochytrium* genus, that are able to produce between 5 and 20% higher EPA than their host counterparts.

Additional bottlenecks include growth standardization and culture regeneration constraints, cost-effective, and scalable extraction processes that minimize oxidation and safety approvals related with regulatory issues (Magoni et al. 2022). Engineering approaches coupled to profound knowledge on the influence of environmental factors on algae physiology and biological activity may play key roles in overcoming these constraints and pave the way to large-scale production and economic competitiveness. A critical analysis on the interdependence of these factors, as well as potential solutions spanning the use of inexpensive substrates, microalgae bioprospecting and adequate strain selection, geographical location, growth enhancement strategies, and minimization of steps required for oil recovery from cells have been covered by Magoni et al (2022). For a more techno-economical analysis on the same influencing factors, see Chauton et al. (2015). From the environmental perspective, a focus on EPA produced from microalgae via heterotrophy is also desirable since studies have shown that the primary energy demand and environmental impacts of PUFA production from microalgae via heterotrophy were significantly lower compared to PUFA produced via photoautotrophy (Togarcheti and Padamati 2021) .

Furthermore, not to be overlooked is also the fact that once all PUFA and remaining lipids have been extracted from the phototrophic or heterotrophic microalgal biomass the remaining cake is still rich in other nutrients (proteins, carbohydrates, vitamins, minerals) or value-added metabolites (natural pigments, carotenoids) which can still be used by food, feed, cosmetic, and agriculture industries (da Silva et al. 2019). Naturally, being able to use the whole microalgal biomass for conversion to high-value products enables a zero-residue generation strategy and the conjugation of various processes may decrease operation costs (Sivaramakrishnan et al. 2022; da Silva et al. 2019). Research and innovation need to continue to pursue efficient continuous production strategies and separation methods with low-energy consumption driving toward increased economic viability and larger production scale of microalgal biorefinery with energy and environmental benefits.
